# Artificial intelligence in genomics: improving detection of fusion genes in soft tissue sarcomas

**DOI:** 10.1097/MS9.0000000000004206

**Published:** 2025-12-01

**Authors:** Muhammad Khizar, Muhammad Zaib, Savera Ejaz, Hasibullah Aminpoor, Hasiba Karimi

**Affiliations:** a Faculty of Medicine, Georgian American University, Manifest Medical Research Co., Tbilisi, Georgia; b Medicine Department, Shaheed Mohtarma Benazir Bhutto Medical College Karachi, Karachi, Pakistan; c Faculty of Medicine, Kabul University of Medical Sciences “Abu Ali Ibn Sina”, Kabul, Afghanistan; d Faculty of Medicine, Bezmialem Vakif University, Istanbul, Turkey

**Keywords:** artificial intelligence, fusion genes, genomics, soft tissue sarcomas

## Abstract

The integration of artificial intelligence (AI) into clinical workflows for soft tissue sarcomas (STS) marks a major step forward in precision oncology. As diagnostic complexity grows, AI-driven genomics – particularly in detecting fusion genes – enhances sensitivity, speed, and accuracy, improving patient stratification and guiding targeted therapies. The convergence of AI with multi-omic datasets, including transcriptomic, epigenomic, and imaging data, offers a holistic view of sarcoma biology, enabling earlier detection and more personalized treatment. Achieving these benefits, however, requires global collaboration, standardized protocols, and continuous clinician education. Ultimately, AI’s transformative potential in STS management depends not only on technological innovation but also on the establishment of robust frameworks for its clinical adoption.

*Dear Editor,*

Soft tissue sarcomas (STS) represent a heterogeneous group of mesenchymal malignancies with variable prognosis and therapeutic responsiveness. A significant subset of STS is driven by specific fusion genes, such as SS18-SSX1 in synovial sarcoma and EWSR1-FLI1 in Ewing sarcoma, which serve as both diagnostic biomarkers and potential therapeutic targets^[1,2]^. Early and accurate detection of these fusions is critical for precise classification, prognostication, and treatment selection. Traditional diagnostic methods, including fluorescence *in situ* hybridization (FISH) and reverse transcription polymerase chain reaction (RT-PCR), are constrained by prior knowledge of fusion partners and may overlook rare or novel rearrangements, highlighting a pressing need for more comprehensive approaches^[3]^.

Recent advances in next-generation sequencing (NGS) technologies have revolutionized the detection of fusion genes in STS. Targeted RNA panels, whole-transcriptome sequencing, and integrative bioinformatic pipelines now enable the identification of both canonical and previously uncharacterized fusions, enhancing diagnostic sensitivity and informing precision oncology strategies^[4]^. However, the increasing complexity and volume of genomic data necessitate computational tools capable of accurate interpretation. AI approaches, including machine learning and deep learning models, have demonstrated remarkable utility in parsing high-dimensional sequencing data, distinguishing driver fusions from passenger alterations, and predicting functional impact^[5,6]^.

Internationally, several countries have spearheaded the integration of AI in sarcoma genomics.

In the United States, consortia such as the Sarcoma Alliance for Research through Collaboration (SARC) are employing AI algorithms to streamline NGS-based diagnostics and facilitate multi-institutional data sharing^[5]^. The United Kingdom has similarly adopted AI-guided workflows at major cancer centers, improving turnaround times and diagnostic precision for rare sarcoma subtypes^[6]^. In Japan, institutions such as the National Cancer Center have combined AI-assisted sequencing with histopathological and radiomic analyses, producing integrative models that refine fusion gene detection and prognostication^[7]^. These international efforts underscore the global potential of AI in enhancing diagnostic accuracy and accelerating translational applications in STS care.

Despite these advances, challenges remain. AI algorithms require large, high-quality datasets and rigorous validation across diverse populations to ensure reliability and generalizability^[2,4]^. Integration into routine clinical workflows necessitates regulatory guidance, standardization of reporting, and education for clinicians and laboratory personnel. Looking forward, the combination of AI with multi-omic datasets, including transcriptomic, epigenomic, and imaging data, holds promise for a comprehensive understanding of sarcoma biology, enabling earlier detection, personalized therapy selection, and improved patient outcomes.

In conclusion, AI-driven genomics represents a transformative tool in the detection of fusion genes in STS. By enhancing the sensitivity, speed, and accuracy of diagnostic workflows, AI has the potential to significantly improve patient stratification and inform targeted therapeutic strategies. Continued international collaboration, methodological refinement, and clinical integration are essential to fully realize the benefits of AI in sarcoma management. We confirm that no AI tools were utilized in the conduct or writing of this manuscript in accordance with the TITAN checklist^[8]^.

## Data Availability

Not applicable for this review.
